# Daily assessments of behavioral economic indices and drinking among rural‐residing adults with at‐risk drinking

**DOI:** 10.1002/jeab.70141

**Published:** 2026-09-07

**Authors:** Devin C. Tomlinson, Erin E. Bonar, Maureen A. Walton, Lewei A. Lin, Inbal Nahum‐Shani, Mary Jannausch, Lara N. Coughlin

**Affiliations:** ^1^ Addiction Center, Department of Psychiatry University of Michigan Ann Arbor MI USA; ^2^ Michigan Innovations in Addiction Care through Research and Education, Department of Psychiatry University of Michigan Ann Arbor MI United States; ^3^ Institute for Social Research University of Michigan Ann Arbor MI United States

**Keywords:** alcohol, alcohol‐free activities, daily surveys, delay discounting, demand

## Abstract

In a secondary analysis among adults with at‐risk drinking who live in rural areas (*N* = 75; 62.7% Female; 98.7% White), we examined the predictive ability of behavioral economic (BE) indices (demand, delay discounting, and proportion of alcohol‐free activities) for next‐day alcohol use via daily surveys across the range of alcohol use severity. These relations were tested using linear mixed‐effects models, including evaluating the interaction of BE indices with alcohol use severity. In unadjusted analyses, proportion of alcohol‐free activities (β [95% CI]: −0.022 [−0.038, −0.006]), demand intensity (i.e., drinks when free; β [95% CI]: 0.267 [0.143, 0.391]), and maximum expenditure (*Omax*, β [95% CI]: 0.036 [0.014, 0.059]) were significant predictors of next‐day alcoholic drinks consumed. In adjusted analyses, we observed a significant interaction between baseline alcohol use severity and intensity of alcohol demand (β [95% CI]: −0.014 [−0.027, −0.001]) and *Omax* (β [95% CI]: 0.005 [0.000, 0.010]) wherein there was a stronger relations between the BE measure and next‐day drinking among lower and higher alcohol use severities, respectively. These results support the inclusion of BE measures in daily survey research to support digital and potentially just‐in‐time adaptive interventions.

## INTRODUCTION

Despite decades of progress in understanding alcohol use and its consequences, we remain limited not by a lack of behavioral economic evidence but by uncertainty about how to apply it in real time to prevent harm and support recovery. To advance the science of alcohol use—and its translation into timely, actionable tools—we must continue to question what we assume about how, when, and why people drink. Behavioral economics offers a powerful lens for doing so, illuminating the moment‐to‐moment decisions that shape health behavior. Warren K. Bickel's pioneering work and mentorship of this new generation of scientists laid the foundation for applying behavioral economics to substance use, demonstrating the relevance of decision‐making processes—like delay discounting—in understanding addiction risk and guiding treatment innovation.

Alcohol use is a significant public health problem. Risky drinking, including binge drinking and heavy drinking, is a risk factor for developing alcohol use disorder (AUD) and other long‐term health consequences such as cancer, heart disease, and liver disease (CDC, 2024). Both alcohol use prevention and treatment interventions are underused; for example, fewer than 10% of individuals with a past‐year AUD received treatment in 2023 (SAMHSA, Center for Behavioral Health Statistics and Quality, 2024). Furthermore, disparities exist in the prevalence of AUD, alcohol‐related harms, and accessing prevention and treatment services for subsets of the population. For example, a recent scoping review reported that rural residence, relative to urban, was associated with an increased risk for hazardous alcohol use and alcohol‐related harms (Friesen et al., 2022). Moreover, individuals in rural locations are less likely to receive care for alcohol use and may experience unique barriers related to care, such as access or cost (Davis & O'Neill, 2022).

Behavioral economics, the study of human behavior and decision making, examines choice preferences around substance use and outcomes among individuals who use substances. Behavioral economic indices include measures such as delay discounting, which is a measure of reward valuation as a function of delay to receipt of the reward (Odum, 2011); alcohol demand (MacKillop & Murphy, 2007), which is a measure of the reinforcing value of alcohol; and time spent on alcohol‐related versus alcohol‐free activities (Murphy et al., 2005). In meta‐analyses, greater delay discounting (Amlung et al., 2017) and greater alcohol demand (Martínez‐Loredo et al., 2021) are associated with alcohol‐related outcomes (e.g., increased severity of alcohol use disorder, heavy drinking). In other work, measures of time spent on alcohol‐free activities show positive associations with reduced alcohol use over time (Acuff et al., 2024; Murphy et al., 2005).

Behavioral economic measures also provide reliable associations with alcohol use across diverse populations (Acuff et al., 2019; Amlung et al., 2017; Kiselica et al., 2016). To date, there is limited evidence suggesting that behavioral economic indices may fluctuate in the context of state and context‐based changes to predict proximal alcohol use, with additional studies needed. For example, higher same‐day alcohol demand intensity is associated with greater subsequent same‐day drinking (Merrill & Aston, 2020). Similarly, higher alcohol demand is associated with cross‐sectional alcohol use and is predictive of alcohol use at the next assessment point, later that day or the next (Motschman et al., 2022). Using within‐person examinations of daily diaries of college students who drink alcohol, greater amounts of time spent on substance‐free activities than usual were associated with consuming less alcohol than usual (Oddo et al., 2024).

Rural populations experience a distinct and, in some cases, widening burden of alcohol‐related harm, including elevated alcohol‐attributable mortality and reduced access to specific treatment services relative to urban populations (Friesen et al., 2022; Spencer et al., 2020). Rural communities also face persistent structural barriers (e.g., workforce shortages, transportation burden, stigma, and limited behavioral health infrastructure) that constrain engagement with substance‐free alternative reinforcers and treatment (Andrilla et al., 2018). These contextual features are directly relevant to behavioral economic theory. Behavioral economic models conceptualize alcohol misuse as emerging from heightened valuation of immediate alcohol reward and reduced access to or valuation of delayed and alternative rewards (Bickel et al., 2014). Rural environments may amplify these mechanisms by limiting access to alternative reinforcers and increasing structural barriers to care, making a behavioral economic framework particularly well suited to examine at‐risk alcohol use in this population.

Furthermore, this project addresses key limitations in the extant behavioral economic literature. Although behavioral economic indicators such as demand and delay discounting are consistently associated with alcohol use and alcohol‐related problems (Amlung et al., 2017; Martínez‐Loredo et al., 2021), much of this work is cross sectional and conducted in urban or college‐based samples. Prospective longitudinal examinations of multiple behavioral economic domains are comparatively limited, and rural populations have been underrepresented in this research. By conducting a longitudinal examination of multiple behavioral economic constructs in an exclusively rural sample, the present study extends prior cross‐sectional findings, evaluates prospective risk pathways, and generates mechanistic data specific to a population with documented structural vulnerability.

In this report, we prospectively examine three commonly used behavioral economic tasks and their association with next‐day alcohol use among participants residing in rural areas who were invited to complete daily ecological momentary assessments (EMAs) as a part of a clinical trial for at‐risk drinking among rural adults (Bayrakdarian et al., 2024). We hypothesized that higher delay discounting rates, higher demand for alcohol, and lower time spent on alcohol‐free activities would be associated with greater next‐day alcohol use. Moreover, we hypothesized that these relations would differ based on an individual's alcohol use severity (measured by the Alcohol Use Disorders Identification Test (AUDIT; Saunders et al., 1993).

## METHODS

The present report is a secondary, exploratory analysis of data from a pilot study (Bayrakdarian et al., 2024), which was approved by an Institutional Review Board and registered at Clinicaltrials.gov.

### Design

The parent project (Bayrakdarian et al., 2024) was a pilot, randomized controlled trial evaluating the feasibility and acceptability of a mobile, behavioral economic intervention for at‐risk alcohol use among rural‐residing adults. Potentially eligible participants were identified through electronic health records at a large academic health care system if they met initial prescreening criteria: (a) being 18 years of age and older with a primary care appointment in the past 2 years and (b) home address zip code in a rural‐designated area (i.e., Rural–Urban Commuting Area or CMS Rural Health Areas). These potentially eligible participants were recruited remotely (i.e., telephone, text, email) to screen for eligibility including (a) AUDIT‐Consumption (AUDIT‐C) score of ≥3 in females or ≥4 in males, an indicator of “at‐risk drinking” (Bradley et al., 2007; Bush et al., 1998) and (b) regular access to an internet‐enabled device. Participants were excluded from the study if they (a) did not understand English, (b) were currently pregnant, (c) were unable to provide informed consent for medical/psychiatric reasons, and/or (d) in current treatment for a substance use disorder. Of note, no pregnant people with at‐risk alcohol use screened during the study.

Recruitment through the EHR was intentional and designed to capture a broad, community‐based sample of people engaged in at‐risk alcohol use rather than a treatment‐seeking subgroup. In rural communities, primary care often serves as the principal point of health care contact due to shortages of specialty behavioral health providers and limited access to SUD treatment services (Larson et al., 2020; Meit et al., 2014). Importantly, rural residents often have a usual care provider at higher rates than urban residents (Kirby & Yabroff, 2020; Agency for Healthcare Research and Quality, 2021). National data indicate the majority of people who may benefit from specialty SUD services do not receive them (SAMHSA, 2025). Thus, EHR‐based identification, including from primary care settings, functions as a population health strategy that reflects real‐world rural health care use patterns and allows proactive identification of people across the continuum of at‐risk drinking, including those not actively seeking treatment. This approach aligns with the national recommendations emphasizing alcohol drinking in primary care (e.g., US Preventive Services Task Force et al., 2018) and enhances external validity by avoiding a treatment‐enriched sample that would systematically exclude many at‐risk rural residents.

Following informed consent, participants were randomized to one of four conditions: episodic future thinking (EFT), volitional choice (VC), EFT and VC, or an enhanced usual care control (EUC). These interventions were hypothesized to reduce alcohol use. As a part of the 2‐week intervention, participants received ecological momentary interventions (EMIs) twice a day from their respective conditions and were asked to complete a survey once daily. The present report evaluates daily survey responses among all participants enrolled in the study.

### Measures

#### Demographics

Demographic characteristics including age, sex at birth, and severity of alcohol use as measured by the AUDIT (Saunders et al., 1993) were collected at baseline.

#### Daily surveys

Daily surveys were sent once per day for the 2‐week intervention period at a participant's preferred time via Qualtrics, and participants had until the end of the day to complete the daily survey (Bayrakdarian et al., 2024). Daily survey items included an assessment of the number of drinks consumed on the previous day in standard drinks and one of three behavioral economic measures: brief assessment of alcohol demand, delay discounting, and an assessment of time spent and enjoyment from alcohol‐related and alcohol‐free activities. The behavioral economic measures were randomized to be assessed daily at a 0.33 probability each day to maintain novelty of the assessment and reduce participant burden (Bayrakdarian et al., 2024).

##### Drinks per day

Participants were asked how many standard drinks they consumed the previous day and entered a number into a textbox as a response (Moody et al., 2018).

##### Proportion of alcohol‐free activities

Participants were asked four questions related to the amount of time spent engaging in activities with and without alcohol as well as how much they enjoyed these activities (Coughlin et al., 2023). The questions were (1) “Think about your day yesterday, how much of your day did you spend doing activities while you were drinking or experiencing the effects of alcohol?” (2) “Overall, how much did you enjoy the activities that included alcohol use yesterday?” (3) “Still thinking about your day yesterday, what percent of the day did you spend doing activities without alcohol use?” and (4) “Overall, how much did you enjoy the alcohol‐free activities you engaged in yesterday?” The questions were answered on a 0‐ to 100‐point scale, where 0 corresponded to “*None*” or “*Not enjoyable*” and 100 corresponded to “*All day*” or “*Very enjoyable*.” To calculate the proportion of time spent on alcohol‐free activities, the amount of time spent on activities without alcohol use was divided by the sum of time spent on activities with and without alcohol use, as described in Bayrakdarian et al. (2024).

##### Delay discounting

The six‐item delay discounting task (i.e., minute discounting task: Koffarnus & Bickel, 2014) was used to assess a participant's *k* value, or the degree to which a participant discounts future rewards. In this task, participant responses estimate their effective delay 50 (ED50), or the delay at which the value of the reinforcer is half of its nominal value, which is equal to 1/*k* (Koffarnus & Bickel, 2014; Yoon & Higgins, 2008). Of the 308 instances of the task, 27 task responses across 15 unique participants indicated the incorrect choice of the sixth attention check item and were not assigned a *k* value, consistent with Koffarnus et al. (2021). Scored participant *k* values were natural‐log transformed for analyses.

##### Brief Assessment of Alcohol Demand

To assess behavioral economic demand, participants answered the three‐item Brief Assessment of Alcohol Demand (BAAD) in the unit of standard drinks with reference to how they were feeling today (Owens et al., 2015). The three items correspond to behavioral economic demand indices of intensity (i.e., standard alcoholic drinks consumed when free), *Omax* (i.e., maximum expenditure on standard drinks in a single session), and breakpoint (i.e., price when consumption is suppressed to zero; Amlung et al., 2015).

### Statistical analysis

Descriptive statistics were performed on demographic data using means and standard deviations or frequencies where appropriate. To determine the association of behavioral economic measures with next‐day drinking, linear mixed‐effect models were used with repeated measures across days within participants. For a visual representation of what data was collected at different points in the daily surveys, please see Supplementary Table 1. Effective sample sizes for models included at least two observations of the behavioral economic measure: proportion of alcohol‐free activities (*n* = 63; 84% of total *N* = 75), delay discounting (*N* = 61; 81.3%), and the BAAD (*n* = 69; 92%). Missing data was treated as missing at random, and data were not imputed. In separate unadjusted models, the behavioral economic measure (i.e., delay discounting rate, proportion of alcohol‐free activities, intensity, *Omax*, and breakpoint) was used to evaluate next‐day drinking with a random effect. Baseline AUDIT score served as a within‐participants random intercept and, in adjusted models, also as a fixed main effects predictor, and it was centered at its baseline study population mean of 7.9189 prior to modeling. Similarly, each behavioral economic measure was initially included as a time‐varying, within‐participant random effect in all models. We tested whether the covariance estimate associated with the random predictor was significant; in models where it was not significant, the predictor appeared only as a fixed effect. In the adjusted models, we tested for an interaction between the fixed effects (i.e., behavioral economic measure and centered baseline AUDIT score); the interaction was removed from the model if not statistically significant. In models with significant interactions, the moderating effect of the centered AUDIT score was examined (Cooper et al., 2023). In a sensitivity analysis, treatment condition was added as a fixed effect to both the unadjusted and adjusted models, where we evaluated an interaction between treatment condition and BE indices; these interactions were not statistically significant and therefore were removed from the models. In main effects analyses, the effect of treatment condition was not significant (see Supplementary Tables 2 and 3). Statistical significance was determined by evaluating the 95% confidence Interval (CI) on estimates. All statistical analyses were performed using SAS version 9.4 (SAS Institute, Cary, NC, USA).

## RESULTS

### Demographics

Table 1 displays the demographic characteristics of the study sample (*n* = 75). Briefly, participants were, on average, 55.2 years old (*SD* = 15.9) and 60% identified as women. The sample was predominantly White (98.7%) and non‐Hispanic/Latinx (93.3%). The majority of participants (66.3%) had a yearly income of less than $75,000. On average, participants had an AUDIT score of 7.9 at baseline (*SD* = 6.8).

**TABLE 1:** Demographic characteristics of the sample.

Baseline characteristic	Mean (*SD*) or *n* (%)
***N***	75 (100%)
**Age in years**	55.2 (15.9)
**Sex at birth**	
Male	28 (37.3%)
Female	47 (62.7%)
**Gender identity**	
Male	28 (37.3%)
Female	45 (60.0%)
Genderqueer/nonconforming	1 (1.3%)
Nonbinary	1 (1.3%)
**Racial identity**	
White	74 (98.7%)
More than one race	1 (1.3%)
**Ethnicity**	
Hispanic/Latino/Latina	5 (6.7%)
Not Hispanic	70 (93.3%)
**Annual income (US$)**	
< $15,000	8 (10.8%)
$15,000–$24,999	7 (9.5%)
$25,000–$34,999	7 (9.5%)
$35,000–$49,999	11 (14.9%)
$50,000–$74,999	16 (21.6%)
$75,000–$99,999	6 (8.1%)
$100,000 +	19 (25.7%)
**AUDIT Score**	7.9 (6.8)

### Unadjusted models

#### Alcohol‐free activities

**FIGURE 1:**
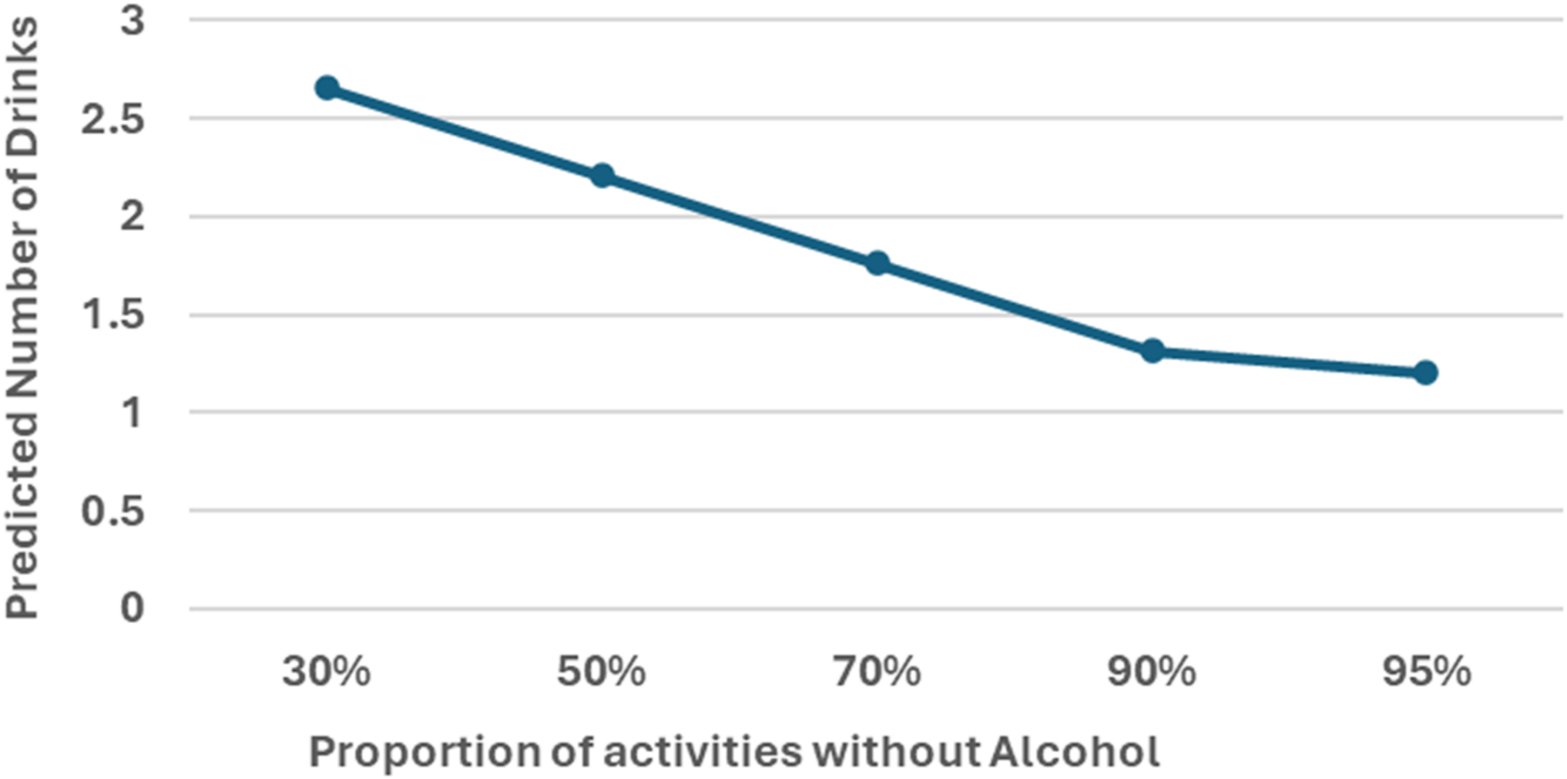
Predicted number of next‐day drinks from the unadjusted model examining the effect of the proportion of alcohol‐free activities on next‐day drinking.

In unadjusted analyses, we report that the proportion of alcohol‐free activities was a significant predictor of next‐day drinking (β [95% CI]: −0.022 [−0.038, −0.006]; Table 2). As the proportion of time spent engaging in activities without alcohol increases, the number of drinks on the next day decreases by approximately 0.02 standard drinks for every unit increase in the proportion of alcohol‐free activities (Table 2; Figure 1).

**TABLE 2:** Unadjusted models of behavioral economic measures predicting next‐day drinking, with a random intercept for alcohol use severity measured by the AUDIT.

	Behavioral economic (BE) predictor of next‐day drinking				
	Proportion of alcohol‐free activities	ln(*k*)	Demand: Intensity	Demand: *Omax*	Demand: Breakpoint
Intercept (95% CI)	3.326 (1.864, 4.788)	1.205 (−0.181,2.592)	0.790 (0.143, 0.391)	0.966 (0.633, 1.299)	1.289 (0.915, 1.633)
Beta: BE measure (95% CI)	−0.022 (−0.038,‐0.006)	−0.062 (−0.281,0.157)	0.267 (0.143, 0.391)	0.036 (0.014, 0.059)	0.019 (−0.027, 0.065)

*Note*: Values of the intercept and β are in units of standard drinks.

#### Delay discounting

In the unadjusted model examining delay discounting rate, we did not observe a significant main effect of ln(*k*) on next‐day drinking (β [95% CI]: −0.062 [−0.281, 0.157]; Table 2).

#### Demand

**FIGURE 2:**
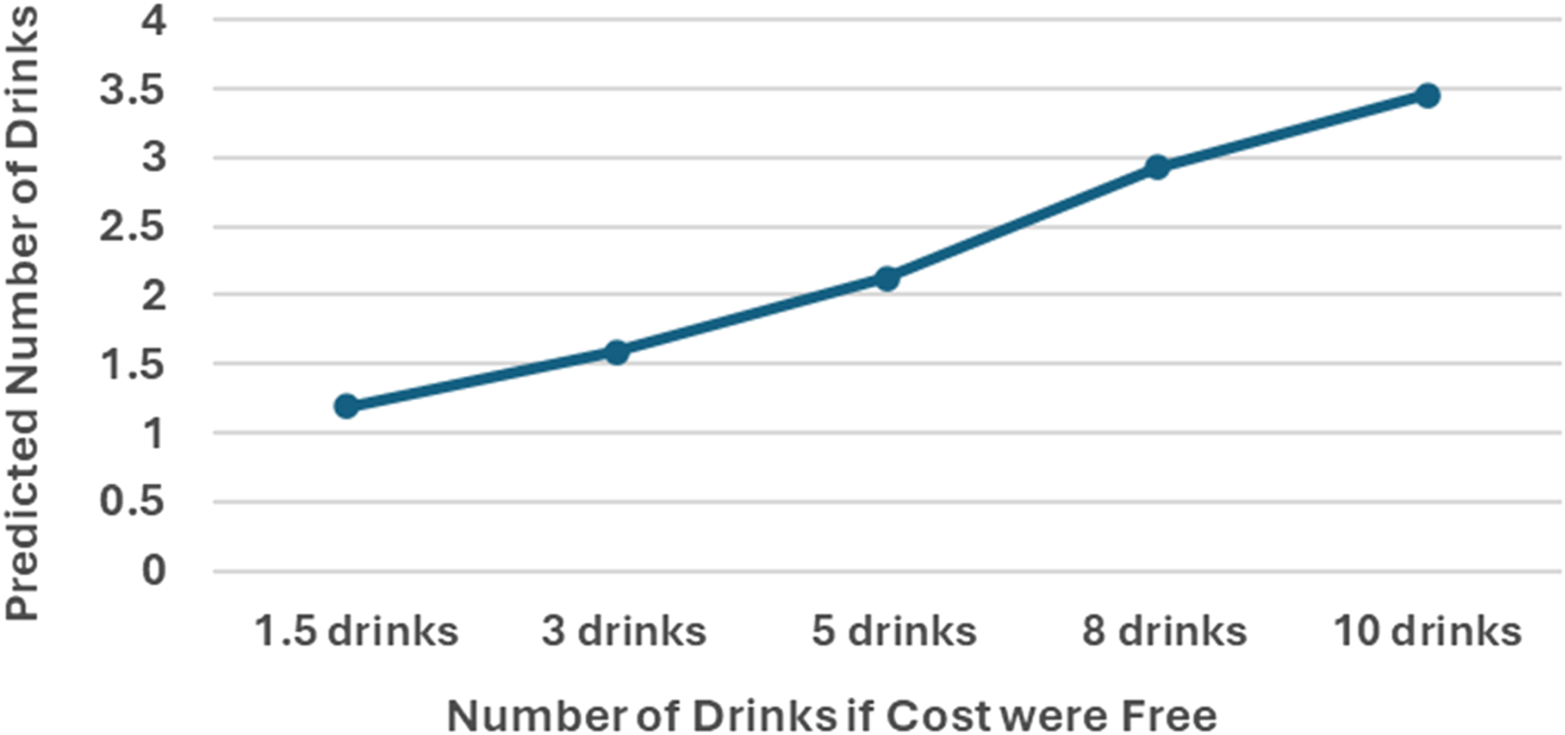
Predicted number of next‐day drinks from the unadjusted model examining the effect of the Intensity item of the Brief Assessment of Alcohol Demand on next‐day drinking.

**FIGURE 3:**
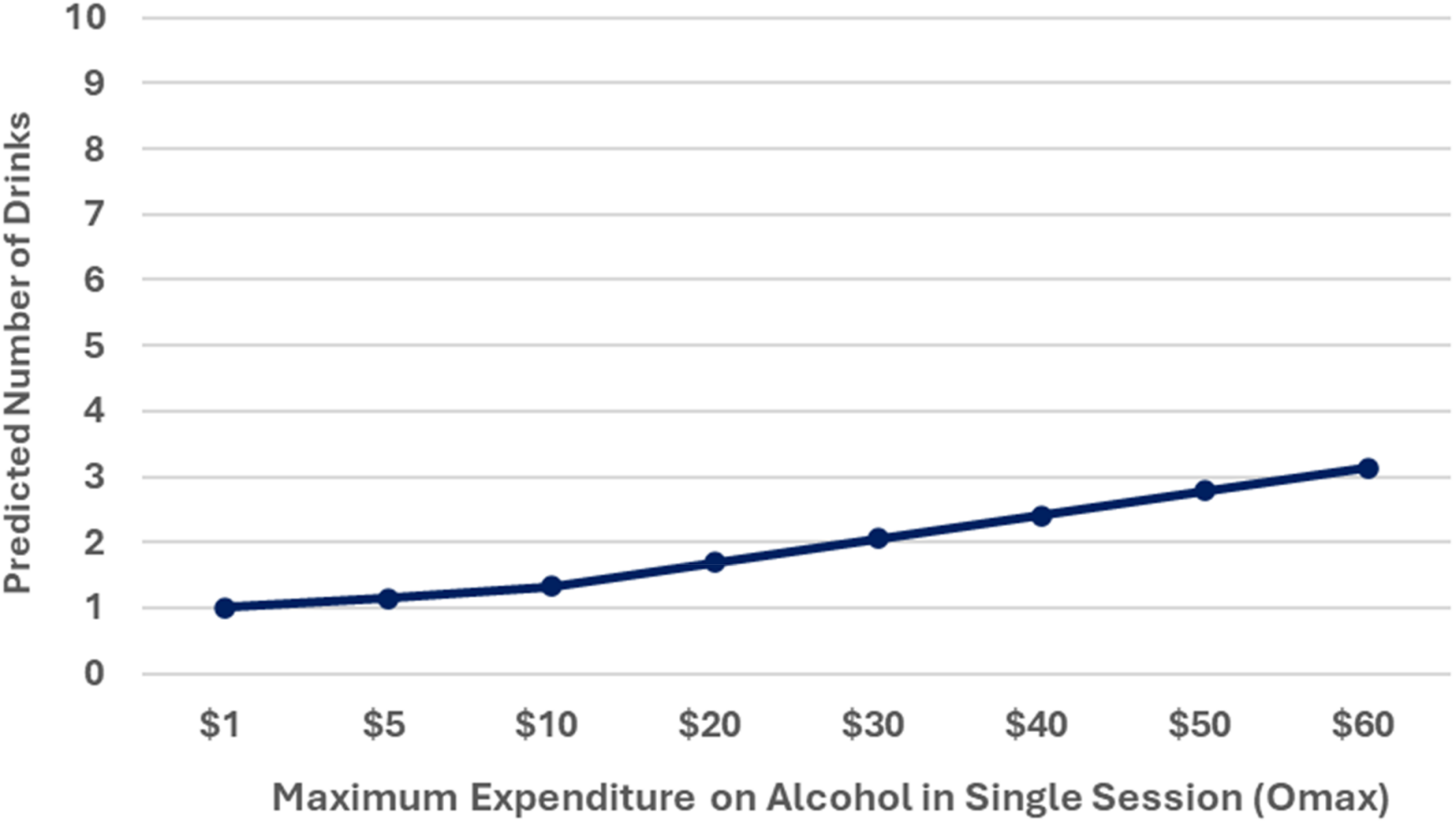
Predicted number of next\day drinks from the unadjusted model examining the effect of the *Omax* item of the Brief Assessment of Alcohol Demand on next‐day drinking.

In unadjusted analyses of the intensity of alcohol demand item of the BAAD, we report that intensity was a significant predictor of next‐day drinking (β [95% CI]: 0.267 [0.143, 0.391]; Table 2). As intensity increases by one unit, the number of drinks on the next day increases by approximately 0.27 standard drinks (Table 2; Figure 2). In unadjusted analyses of *Omax*, we report *Omax* as a significant predictor of next‐day drinking (β [95% CI]: 0.036 [0.014, 0.059]; Table 2; Figure 3). As *Omax* increases, the number of drinks on the next day increases by approximately 0.04 standard drinks for every unit increase in *Omax* (Table 2; Figure 3). We did not, however, observe a statistically significant effect of breakpoint on next‐day drinking (β [95% CI]: 0.019 [−0.027, 0.065]; Table 2).

### Adjusted nodels

#### Alcohol‐free activities

In adjusted analyses evaluating the effect of both proportion of alcohol‐free activities and baseline alcohol use severity, as measured by the AUDIT, we observed a significant effect of baseline alcohol use severity (β [95% CI]: 0.211 [0.128, 0.294]; Table 3; Supplementary Table 4). We did not observe a significant main effect of the proportion of alcohol‐free activities after adjusting for centered baseline AUDIT (β [95% CI]: −0.014 [−0.031, 0.002]; Table 3). We did not observe a statistically significant interaction between alcohol use severity and the proportion of alcohol‐free activities (Table 3).

**TABLE 3:** Adjusted models of behavioral economic measures predicting next‐day drinking and baseline alcohol use severity as measured by the AUDIT, with a random intercept for AUDIT scores.

	Behavioral economic (BE) predictor of next‐day drinking				
	Proportion of alcohol‐free activities	ln(*k*)	Demand: intensity	Demand: *Omax*	Demand: Breakpoint
Intercept (95% CI)	2.998 (1.519, 4.478)	1.272 (−0.62, 2.606)	1.086 (0.753, 1.419)	1.222 (0.882, 1.561)	1.548 (1.191, 1.905)
β: BE measure (95% CI)	−0.014 (−0.031, 0.002)	−0.103 (−0.316,0.109)	0.291 (0.173, 0.409)	0.041 (0.019, 0.062)	0.030 (−0.012, 0.072)
β: AUDIT (95% CI)	0.211 (0.128, 0.294)	0.222 (0.121, 0.322)	0.190 (0.116, 0.265)	0.124 (0.035, 0.212)	0.197 (0.127, 0.267)
β: Interaction (95% CI)	‐	‐	−0.014 (−0.027, −0.001)	0.005 (0.000, 0.010)	‐

*Note*: Values of the intercept and β are in units of standard drinks.

#### Delay discounting

In the adjusted model after adjusting for centered baseline alcohol use severity, ln(*k*) was not a significant predictor of next‐day drinking (β[95% CI]: −0.103 [−0.316, 0.109]; Table 3; Supplementary Table 4). Alcohol use severity was a significant predictor of next‐day drinking (β [95% CI]: 0.222 [0.121, 0.322]; Table 3). Although tested for, we did not observe a statistically significant interaction between alcohol use severity and rate of delay discounting (Table 3).

#### Demand

**FIGURE 4:**
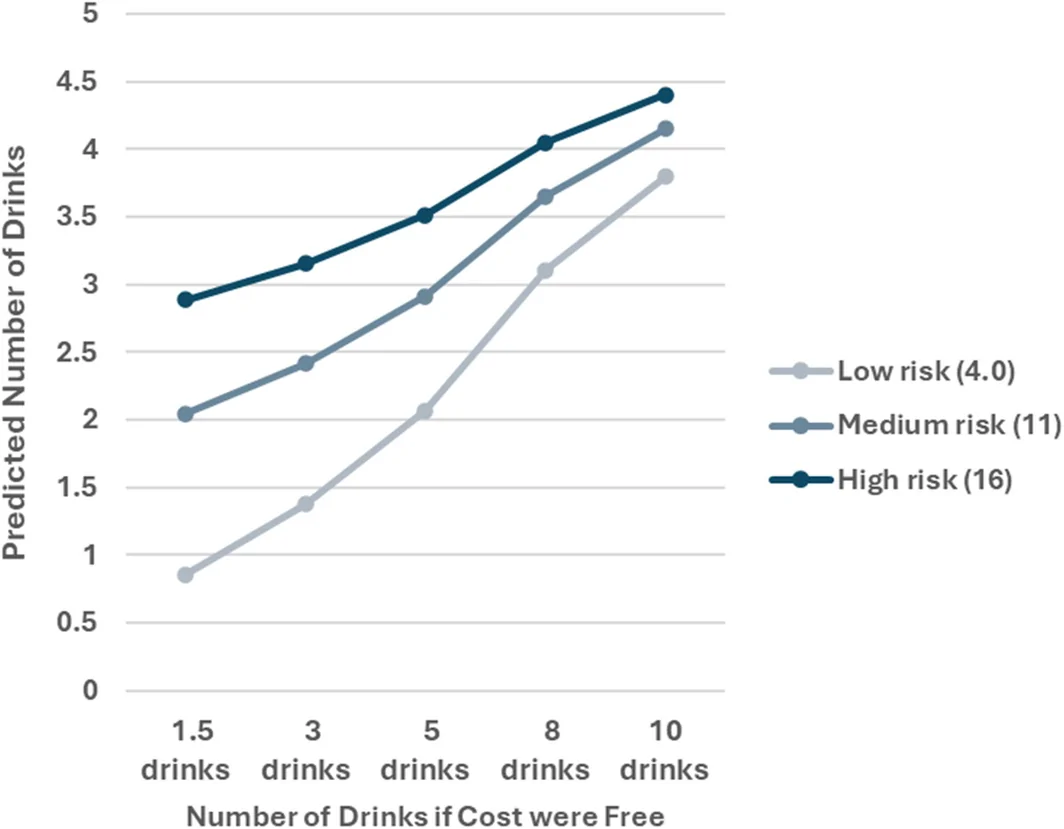
Predicted number of next‐day drinks from the adjusted model examining the effect of the interaction of the Intensity item of the Brief Assessment for Alcohol Demand and alcohol use severity, measured by AUDIT, on next‐day drinking. AUDIT scores indicating low risk (4), medium risk (11), and high risk (16) for an alcohol use disorder are shown in the plot.

In adjusted analyses evaluating both intensity and baseline alcohol use severity, we observed a significant interaction between centered baseline alcohol use severity and intensity (β [95% CI]: −0.014 [−0.027, −0.001]; Table 3; Supplementary Table 4; Figure 4) as well as main effects of both intensity (β [95% CI]: 0.291 [0.173, 0.409]; Table 3) and centered baseline alcohol use severity (β [95% CI]: 0.190 [0.116, 0.265]; Table 3). We observed that next‐day drinking is a function of both baseline alcohol use severity and intensity; the relation between alcohol use severity and intensity is stronger among individuals with lower alcohol use severities (Figure 4). Specifically, we observe that for a one‐unit increase in intensity the number of next‐day drinks increases by 0.345 when an individual's alcohol use severity is low, 0.247 drinks when alcohol use severity is moderate, and 0.178 drinks when their alcohol use severity is high.

**FIGURE 5:**
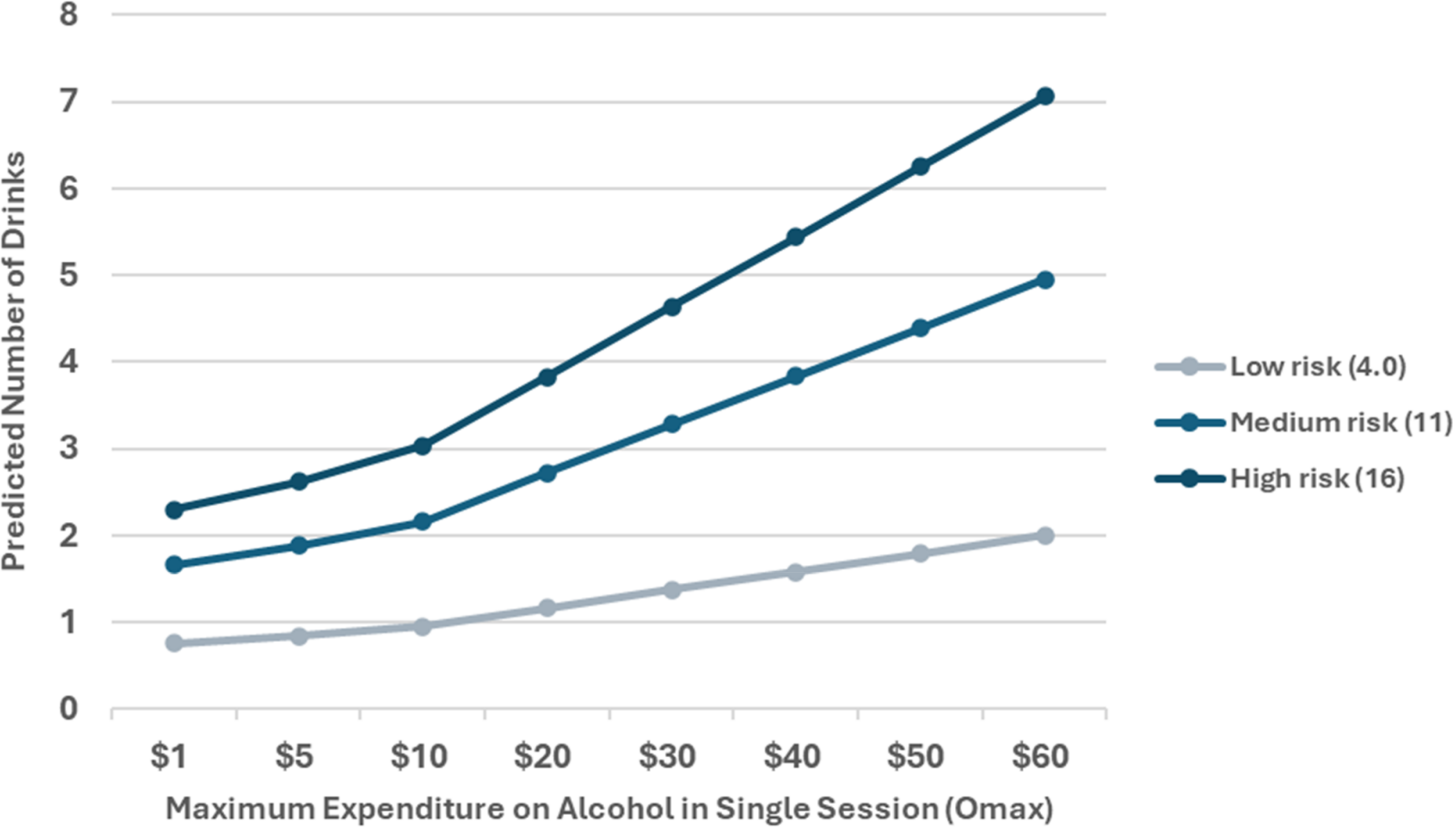
Predicted number of next‐day drinks from the adjusted model examining the effect of the interaction of the *Omax* item of the Brief Assessment for Alcohol Demand and alcohol use severity, measured by AUDIT, on next‐day drinking. AUDIT scores indicating low risk (4), medium risk (11), and high risk (16) for an alcohol use disorder are shown in the plot.

When evaluating *Omax* in adjusted analyses, we observed a statistically significant interaction between an individual's centered alcohol use severity and *Omax* (β [95% CI]: 0.005 [0.000, 0.010]; Table 3; Supplementary Table 4; Figure 5). Moreover, we observed statistically significant main effects of *Omax* (β [95% CI]: 0.041 [0.019, 0.062]; Table 3) and centered alcohol use severity (β [95% CI]: 0.124 [0.035, 0.212]; Table 3). We observed that next‐day drinking is a function of both baseline alcohol use severity and *Omax*; the relation between alcohol use severity and Omax is stronger among individuals with higher alcohol use severities (Figure 5). Specifically, we observe that for a one‐unit increase in *Omax* the number of next‐day drinks increases by: 0.021 when an individual's alcohol use severity is low, 0.056 drinks when alcohol use severity is moderate, and 0.080 drinks when their alcohol use severity is high.

In adjusted analyses evaluating breakpoint, we did not observe a statistically significant interaction between breakpoint and alcohol use severity. We did not observe a statistically significant main effect of breakpoint (β [95% CI]: 0.030 [−0.012, 0.072]; Table 3; Supplementary Table 4); however, we did observe a main effect of alcohol use severity (β [95% CI]: 0.198 [0.127, 0.267]; Table 3).

## DISCUSSION

Among a sample of adults screening positive for at‐risk drinking and residing in rural areas, we evaluated behavioral economic indices measured via daily surveys and their ability to predict next‐day alcohol use. Our results support that on a given day, for an individual, the greater proportion of time spent doing alcohol‐free activities, the lower their demand intensity for alcohol, or the lower their demand *Omax*, the fewer drinks they consumed the following day. Our results did not support daily delay discounting rate, ln(*k*), or breakpoint, as being predictive of next‐day alcohol consumption.

Although there is limited literature on the utility of alcohol demand to predict subsequent drinking, our findings are consistent with Motschman et al. (2022) who found higher demand (i.e., intensity, *Omax*) was associated with greater odds of drinking occurrence or continuation at the following EMA prompt in the natural environment. We did not find, however, an association of breakpoint and next‐day drinking, inconsistent with Motschman et al. (2022). One possible explanation for this null result is that breakpoint is less sensitive than intensity and *Omax* in assessments of relationships with substance use (Zvorsky et al., 2019).

We also report a significant interaction between baseline alcohol use severity and both alcohol demand intensity and alcohol demand *Omax*. That is, individuals with higher baseline alcohol use severity and higher demand intensity or *Omax* have a higher predicted number of next‐day drinks than do either individuals with lower baseline alcohol use severity or lower demand intensity or *Omax*. Specifically, there was a stronger relation between the BE measure (i.e., intensity or *Omax*) and next‐day drinking among lower and higher alcohol use severities, respectively. These results support the inclusion of intensity or *Omax* of alcohol demand in ecological momentary assessment research assessing risk for proximal alcohol use. Assessment of the proportion of substance‐free activities as a brief survey item is a novel application of measures assessing substance‐related and substance‐free reinforcement, although a systematic review on reinforcement measures also suggested the utility of this novel assessment (Acuff et al., 2019).

Literature examining ecological assessment of delay discounting and substance use is limited, especially in the examination of alcohol use. Previous reports have examined delay discounting using ecological assessments in relation to substance use (Luken et al., 2024; Xu et al., 2022), with Luken et al. (2024) reporting no association between ecological momentary assessment measures of delay discounting and alcohol use or alcohol craving. Similar to Luken et al. (2024), we report that delay discounting rate assessed daily using the six‐item adjusting delay task was not a significant predictor of next‐day alcohol consumption. One potential explanation for this null result is that delay discounting rate is stable over long periods and perhaps not as well suited as a time‐varying behavioral economic index of near‐term alcohol use risk in daily assessments over 30 days (Strickland et al., 2023).

Inclusion of tasks rather than questions regarding retrospective substance use may limit assessment reactivity, or the observation that measurement of psychological constructs can change individuals' thoughts, feelings, and behaviors (French & Sutton, 2010). The behavioral economic indices examined herein may provide potential benefits to inform digital interventions without directly asking about recent alcohol use. Future directions for this line of work may include just‐in‐time adaptive interventions (JITAIs) that provide support for individuals based on their state and context (Nahum‐Shani et al., 2018), which based on our findings could include time spent on substance‐free activities and alcohol demand characteristics.

The study is not without limitations that should be acknowledged. First, data in this analysis are from a pilot study focused on acceptability and feasibility, and these results are exploratory. Future research should replicate these analyses with varied samples to determine consistency of findings. Second, the sample is largely White and non‐Hispanic/Latinx, consistent with demographic data of rural Michigan, where 95% of individuals living in rural areas identified as White (Citizens Research Council of Michigan, 2018). These distributions limit the generalizability of our findings to other samples. Given the modest sample size and the pilot nature of the parent trial, the racial representation is generally within expectations and may be reasonably representative of rural Michigan. Future investigations should explore the relation of behavioral economic indices and alcohol use at a daily level among a larger and more diverse sample. Third, the analysis assesses within‐subject variation of behavioral economic indices in relation to next‐day alcohol consumption; however, future investigations should evaluate these relations across diverse patient populations and over longer time frames to continue to understand how behavioral economic indices change across differing states and traits. Fourth, as BE indices were measured once per day, we were unable to examine the effects of delay discounting and relative reinforcing efficacy reinforcer pathology. Investigations with a greater frequency of assessments should investigate these relations. Moreover, other demographic characteristics may moderate the relations between behavioral economic indices and next‐day drinking; future examinations, particularly in larger samples to enhance power, may consider other moderators, such as income or treatment conditions. We note that future investigations may consider replicating these pilot, hypothesis‐generating findings in other samples to assess for generalizability of these reported relationships between behavioral economic indices and drinking at the daily level.

## CONCLUSIONS

We report behavioral economic indices, alcohol demand intensity, *Omax*, and proportion of time spent on alcohol‐free activities, are significantly able to predict next‐day alcohol consumption among adults with at‐risk drinking who live in rural areas. These results support the inclusion of behavioral economic measures in EMA future research as potential considerations for alternative assessment items for alcohol consumption as well as more importantly for delivery of JITAIs.

## AUTHOR CONTRIBUTIONS

D.C. Tomlinson contributed to the conceptualization, writing the original manuscript draft, and review/revisions. E.E. Bonar, M.A. Walton, L.A. Lin, and I. Nahum‐Shani all contributed to the investigation and review/revisions of the manuscript. M. Jannausch contributed to the manuscript through formal analysis, visualization, and manuscript review editing. L.N. Coughlin contributed to the manuscript through conceptualization, investigation, resources, review/editing, supervision, and funding acquisition.

## CONFLICT OF INTEREST STATEMENT

The authors do not have any disclosures to report.

## ETHICS APPROVAL

This study was approved by an Institutional Review Board and registered at Clinicaltrials.gov.

## Supporting information


**Supplementary Table 1** Visual representation of timepoints and items assessed in the ecological momentary assessments.
**Supplementary Table 2**. Sensitivity analysis of unadjusted models of behavioral economic measures and next day drinking with a random intercept for alcohol use severity measured by the AUDIT. Treatment condition is specified as a fixed effect.
**Supplementary Table 3**. Sensitivity analysis of adjusted models of behavioral economic measures and next day drinking and baseline alcohol use severity as measured by the AUDIT with a random intercept for AUDIT scores. Treatment condition is specified as a fixed effect.
**Supplementary Table 4**. Table of covariance parameter estimates from adjusted models.

## Data Availability

The data that support the findings of this study are openly available in NIMH Data Archive at https://nda.nih.gov/edit_collection.html?id=3958, reference number 3958.
