# Supplementary material for: Daily assessments of behavioral economic indices and drinking among rural‐residing adults with at‐risk drinking
Source: J Exp Anal Behav. 2026 Sep 7;126(2):e70141. doi: 10.1002/jeab.70141 (PMC13548064; doi:10.1002/jeab.70141)
Supplement: Supplementary file 1 [file JEAB-126-0-s001.docx]

**Supplementary Material**

**Supplementary Table 1.** Visual representation of timepoints and items assessed in the ecological momentary assessments.

| **Timepoint** | **t-2** | **t-1** | **t** |
| --- | --- | --- | --- |
| **Behavioral Economic Measure** | Demand | Proportion of Alcohol-Free Activities | Previous Day Drinks |
|  | Delay discounting |  |  |

**Supplementary Table 2.** Sensitivity analysis of unadjusted models of behavioral economic measures and next day drinking with a random intercept for alcohol use severity measured by the AUDIT. Treatment condition is specified as a fixed effect.

|  | **Behavioral Economic (BE) Predictor of Next Day Drinking** | | | | |
| --- | --- | --- | --- | --- | --- |
|  | **Proportion of Alcohol-Free Activities** | **ln(*k*)** | **Demand:**  **Intensity** | **Demand: Omax** | **Demand: Breakpoint** |
| **Intercept**  **(95% CI)** | 3.858  (2.199, 5.518) | 0.930  (-0.660, 2.520) | 0.906  (0.284, 1.523) | 1.049  (0.376, 1.723) | 1.411  (0.696, 2.126) |
| **β: BE measure**  **(95% CI)** | -0.025  (-0.042,-0.008) | -0.067  (-0.288, 0.154) | 0.267  (0.140, 0.394) | 0.036  (0.013, 0.059) | 0.017  (-0.031, 0.065) |
| **β: Treatment Condition** |  |  |  |  |  |
| **EFT**  **(95% CI)** | -0.447  (-1.390, 0.495) | 0.127  (-1.195, 1.449) | -0.093  (-0.865, 0.680) | -0.098  (-0.925, 0. 730) | -0.179  (-1.007, 0. 649) |
| **EFT/VCT**  **(95% CI)** | -0.008  (-0.920, 0.903) | 0.642  (-0.639, 1.922) | -0.075  (-0.835, 0.685) | -0.047  (-0.874, 0.779) | -0.178  (-0.968, 0.612) |
| **VCT**  **(95% CI)** | -0.567  (-1.478, 0.342) | 0.158  (-1.119, 1.435) | -0.270  (-1.035, 0.496) | -0.164  (-0.978, 0.650) | -0.051  (-0.843, 0.741) |
| **Control** | (Referent) | (Referent) | (Referent) | (Referent) | (Referent) |

**Supplementary Table 3.** Sensitivity analysis of adjusted models of behavioral economic measures and next day drinking and baseline alcohol use severity as measured by the AUDIT with a random intercept for AUDIT scores. Treatment condition is specified as a fixed effect.

|  | **Behavioral Economic (BE) Predictor of Next Day Drinking** | | | | |
| --- | --- | --- | --- | --- | --- |
|  | **Proportion of Alcohol Free Activities** | **ln(*k*)** | **Demand:**  **Intensity** | **Demand: Omax** | **Demand: Breakpoint** |
| **Intercept**  **(95% CI)** | 3.340  (1.739, 4.940) | 0.918  (-0.597, 2.432) | 1.139  (0.553, 1.725) | 1.272  (0.664, 1.880) | 1.594  (0.959, 2.228) |
| **β: BE measure**  **(95% CI)** | -0.015  (-0.032, 0.001) | -0.105  (-0.319, 0.109) | 0.278  (0.155, 0.402) | 0.041  (0.019, 0.063) | 0.031  (-0.014, 0.075) |
| **β: AUDIT (95% CI)** | 0.207  (0.124, 0.290) | 0.225 (0.121, 0.329) | 0.192  (0.115, 0.269) | 0.124  (0.033, 0.216) | 0.199  (0.127, 0.271) |
| **β: Treatment Condition** |  |  |  |  |  |
| **EFT**  **(95% CI)** | -0.476  (-1.345, 0.393) | 0.253  (-0.964, 1.470) | 0.051  (-0.646, 0.748) | -0.004  (-0.726, 0.717) | -0.002  (-0.722, 0.726) |
| **EFT/VCT**  **(95% CI)** | -0.119  (-0.949, 0.711) | 0.654  (-0.514, 1.822) | -0.051  (-0.734, 0.633) | -0.102  (-0.816, 0.611) | -0.177  (-0.866, 0.512) |
| **VCT**  **(95% CI)** | -0.490  (-1.324, 0.344) | 0.416  (-0.762, 1.594) | -0.115  (-0.801, 0.570) | -0.084  (-0.789, 0.621) | -0.006  (-0.688, 0.700) |
| **Control** | (Referent) | (Referent) | (Referent) | (Referent) | (Referent) |
| **β: BE x AUDIT Interaction**  **(95% CI)** | - | - | -0.014  (-0.027, -0.001) | 0.005  (0.000, 0.010) | - |

**Supplementary Table 4.** Table of covariance parameter estimates from adjusted models.

| **Model: Proportion of Substance Free Activities** | | | | | |  |
| --- | --- | --- | --- | --- | --- | --- |
| **Cov Parm** | **Subject** | **Estimate** | **Standard**  **Error** | **Z Value** | **Prob > Z** |  |
| AUDIT | ID (random) | 0.022 | 0.019 | 2.34 | 0.010 |  |
| AR(1) | ID (repeated day) | 0.294 | 0.094 | 3.12 | 0.002 |  |
| Residual |  | 4.241 | 0.383 | 11.06 | <0.0001 |  |
| **Model: Delay discounting (ln(k))** | | | | | |  |
| **Cov Parm** | **Subject** | **Estimate** | **Standard**  **Error** | **Z Value** | **Prob > Z** |  |
| AUDIT | ID (random) | 0.034 | 0.016 | 2.19 | 0.014 |  |
| AR(1) | ID (repeated day) | 0.698 | 0.056 | 12.43 | <0.0001 |  |
| Residual |  | 4.245 | 0.538 | 7.89 | <0.0001 |  |
| **Model: Intensity** | | | | | |  |
| **Cov Parm** | **Subject** | **Estimate** | **Standard**  **Error** | **Z Value** | **Prob > Z** |  |
| AUDIT | ID (random) | 0.017 | 0.008 | 2.17 | 0.015 |  |
| Intensity | ID (random) | 0.030 | 0.017 | 1.73 | 0.042 |  |
| AR(1) | ID (repeated day) | -0.183 | 0.094 | -1.95 | 0.051 |  |
| Residual |  | 2.307 | 0.215 | 10.71 | <0.0001 |  |
| **Model: Omax** | | | | | |  |
| **Cov Parm** | **Subject** | **Estimate** | **Standard**  **Error** | **Z Value** | **Prob > Z** |  |
| AUDIT | ID (random) | 0.020 | 0.009 | 2.13 | 0.017 |  |
| Omax | ID (random) | 0.001 | 0.001 | 1.77 | 0.038 |  |
| AR(1) | ID (repeated day) | -0.152 | 0.093 | -1.64 | 0.102 |  |
| Residual |  | 2.368 | 0.223 | 10.61 | <0.0001 |  |
| **Model: Breakpoint** | | | | | |  |
| **Cov Parm** | **Subject** | **Estimate** | **Standard**  **Error** | **Z Value** | **Prob > Z** |  |
| AUDIT | ID (random) | 0.022 | 0.009 | 2.3 | 0.011 |  |
| Day | ID (repeated day) | 2.735 | 0.243 | 11.26 | <0.0001 |  |
